# Intractable Nausea as the Initial Presentation of Neuromyelitis Optica

**DOI:** 10.7759/cureus.103669

**Published:** 2026-02-15

**Authors:** Shu Jun Tan

**Affiliations:** 1 Internal Medicine, Raffles Hospital, Singapore, SGP

**Keywords:** autoimmune neurology, bilateral blurring of vision, nausea and vomiting, neuromyelitis optica spectrum disorder, wernicke encephalopathy

## Abstract

Neuromyelitis optica spectrum disorder (NMOSD) is an autoimmune condition of the central nervous system, primarily involving the optic nerves and spinal cord. Patients typically present with visual disturbance, limb weakness or sensory deficits, and bladder dysfunction. However, atypical and nonspecific presentations may occur, including persistent nausea and vomiting due to involvement of the area postrema.

We report the case of a 26-year-old female patient who presented with a one-month history of intractable vomiting, associated with epigastric discomfort (worse prior to vomiting), reduced appetite, and significant weight loss of approximately 7-8 kg over one month. She had been admitted two times prior to presentation, with extensive investigations that were unremarkable.

During admission, she also reported blurred vision and was noted to have horizontal nystagmus. Brain MRI demonstrated areas of altered signal intensity involving the brainstem and cervicomedullary junction, along the floor of the fourth ventricle, initially raising concern for Wernicke’s encephalopathy. She received intravenous thiamine without clinical improvement. Further evaluation, including whole-spine MRI and autoimmune serology, revealed positivity for aquaporin-4 (AQP4) IgG antibodies, confirming NMOSD. She was treated with intravenous methylprednisolone and tocilizumab, resulting in marked symptomatic improvement.

This case highlights NMOSD as an important and often under-recognized cause of intractable nausea and vomiting, which may delay diagnosis and treatment. A thorough neurological history and examination are essential in patients with persistent unexplained vomiting to ensure timely recognition and initiation of appropriate therapy.

## Introduction

Vomiting is a common and nonspecific symptom that is most frequently attributed to gastrointestinal, metabolic, or infectious etiologies [1]. Consequently, neurological causes are often overlooked, particularly in the absence of early focal neurological signs [2]. This may result in repeated hospital admissions, extensive gastrointestinal investigations, and delays in diagnosis. However, persistent or intractable vomiting can represent an atypical manifestation of central nervous system pathology, especially when associated with evolving neurological deficits. The emergence of features such as nystagmus [3], sensory impairment, weakness, or gait instability should prompt urgent neurological evaluation and appropriate neuroimaging. Early recognition of these red flag signs is critical, as timely diagnosis allows for the prompt initiation of disease-modifying therapy, which may significantly improve neurological outcomes and reduce long-term disability [4].

From a neurological perspective, vomiting may arise from relatively benign conditions such as migraine or vestibular disorders, but it may also indicate more serious pathology requiring urgent intervention, including raised intracranial pressure or disorders affecting the lower brainstem. In particular, involvement of the area postrema located in the dorsal medulla can lead to intractable nausea and vomiting [5].

Neuromyelitis optica spectrum disorder (NMOSD) is an autoimmune inflammatory condition of the central nervous system that predominantly affects the optic nerves and spinal cord, with less frequent involvement of the brainstem [6]. Patients classically present with optic neuritis, manifesting as acute visual loss or painful eye movements, and transverse myelitis, which may result in pain, weakness, sensory disturbances, and bowel or bladder dysfunction. Brainstem involvement, especially affecting the area postrema, may cause intractable hiccups, nausea, and vomiting, while hypothalamic involvement has been associated with hypersomnolence or narcolepsy [7].

Our case highlights an uncommon but clinically important presentation of NMOSD, emphasizing the need to consider central nervous system pathology in patients with persistent vomiting and evolving neurological features. Documenting this case aims to raise awareness of area postrema syndrome as a diagnostic clue, reduce delays in diagnosis, and underscore the importance of early immunotherapy in improving neurological outcomes.

## Case presentation

A 26-year-old woman presented to our institution in March 2025 with a one-month history of persistent nausea and vomiting, which had begun in February 2025 and was associated with generalized weakness. Her symptoms initially started with one week of nausea, followed by nonbilious, nonbloody vomiting. The vomiting was nonprojectile, involved small volumes of fluid, occurred predominantly at night, and was associated with epigastric discomfort. She denied fever, diarrhea, constipation, or other changes in bowel habits. There was no history of headache, visual disturbance, or focal neurological symptoms at initial presentation. She also reported no recent travel or sick contacts.

Prior to this admission, she had two previous hospitalizations during which she underwent extensive investigations, including abdominal imaging and upper gastrointestinal endoscopy, and was diagnosed with dyspepsia. Over the preceding month, she also reported anorexia and unintentional weight loss of approximately 7-8 kg.

She had no significant past medical history. On physical examination, she was afebrile, and her abdomen was soft and nontender, with no organomegaly or other abnormal findings. Neurological examination was initially normal on presentation.

Laboratory investigations were performed to evaluate for systemic causes (Table 1). There was no evidence of infection. Electrolyte levels were within normal limits, except for mild hypokalemia attributed to ongoing vomiting. Renal, hepatic, and thyroid function tests were normal.

**Table 1:** Laboratory investigations ALT: alanine transaminase; SGPT: serum glutamic-pyruvic transaminase; AST: aspartate aminotransferase; SGOT: serum glutamic-oxaloacetic transaminase

Parameter	Results	Units	References
White blood cell	5.22	10^9^/L	4-11
Neutrophil	46.0	%	40-75
Lymphocytes	45.0	%	20-45
Monocytes	9.0	%	2-10
Eosinophils	0.0	%	0-6
Basophil	0.0	%	0-1
Hemoglobin	13.4	g/dL	11.5-16
Platelets	239	10^9^/L	140-450
Glucose	5.1	mmol/L	3.3-7 (fasting)
Potassium	3.2	mmol/L	3.3-5.1
Sodium	135	mmol/L	135-150
Calcium	9.7	mg/dL	8.4-10.2
Magnesium	0.91	mmol/L	0.65-1.05
Phosphate	4.1	mg/dL	2.7-4.5
Chloride	96	mmol/L	96-109
Bicarbonate	20	mmol/L	21-32
Urea	30	mg/dL	10-50
Creatinine	0.64	mg/dL	0.50-1.60
Bilirubin, total	0.8	mg/dL	<1.5
Total protein	8.6	g/dL	6.0-8.3
Albumin	4.6	g/dL	3.4-5.1
Globulin	4.0	g/dL	1.9-3.6
Alb/Glo ratio	1.1		1.2-2.5
Alkaline phosphatase	43	U/L	39-117
AST/SGOT	22	U/L	Up to 35
ALT/SGPT	19	U/L	Up to 35
GGT	26	U/L	7-32
C-reactive protein	<0.6	mg/L	<5.0
Amylase, serum	98	U/L	28-100
Thyroid-stimulating hormone (TSH)	1.38	µIU/mL	0.27-4.20
Free T4	1.57	ng/dL	0.93 - 1.71
B12	483	pmol/L	145-569
Folate	13.50	nmol/L	8.83-60.80

**Figure 1:**
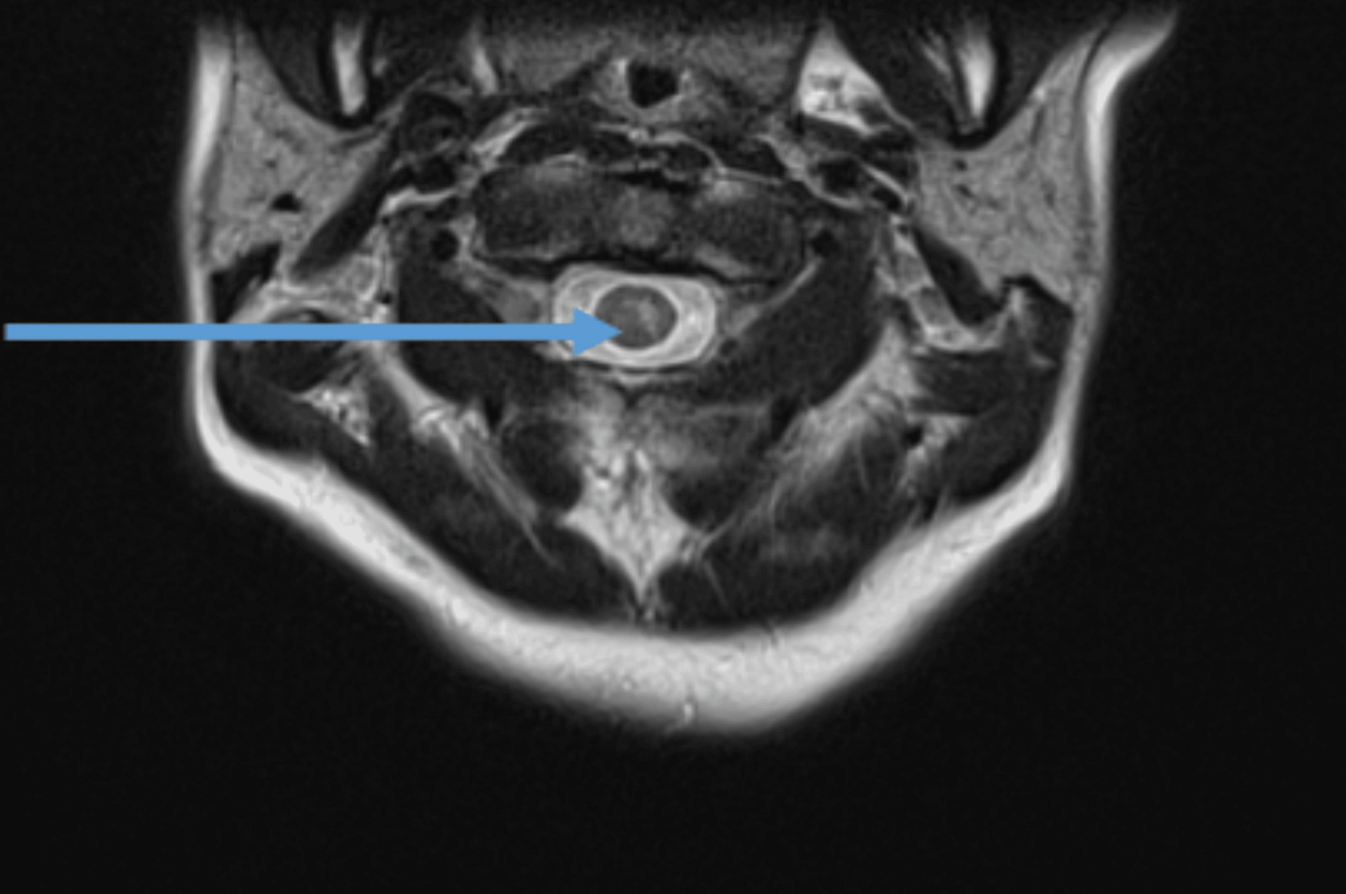
MRI brain: areas of altered signal-T2 hyperintensity with possible restricted diffusion in the brainstem and cervicomedullary junction, along the floor of the fourth ventricle MRI: magnetic resonance imaging

Subsequently, three days into her hospitalization, she reported blurring of vision and an unsteady gait. Neurological examination revealed horizontal nystagmus, impaired proprioception in the right foot, and bilateral lower limb weakness with Medical Research Council (MRC) grade 4-/5 power, more pronounced on the right. Sensory examination demonstrated reduced distal sensation in the digits of both upper limbs. She also had impaired standing balance. Based on these findings, the initial differential diagnoses included Wernicke’s encephalopathy, multiple sclerosis (MS), NMOSD, and myelin oligodendrocyte glycoprotein antibody-associated disease (MOGAD). An MRI brain with contrast was performed (Figure 1).

**Figure 2:**
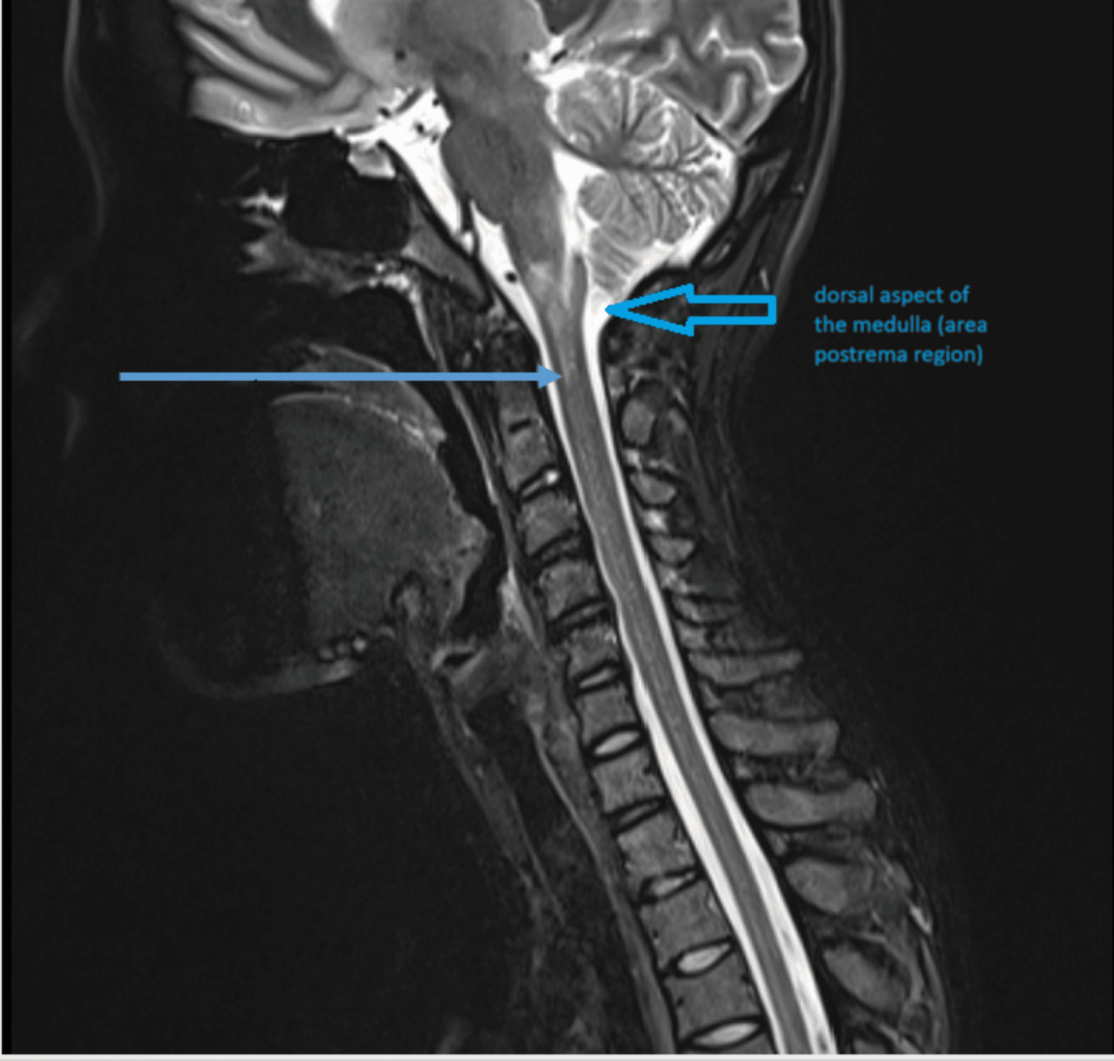
MRI spine: T2 hyperintense signal across the cervical medullary junction measuring nearly 2.7 cm in the craniocaudal extent centered along the floor of the fourth ventricle and in the central cord MRI: magnetic resonance imaging

An MRI cervical spine was performed (Figure 2).

With concerns of transverse myelitis noted on the MRI, an autoimmune screen was performed (Table 2).

**Table 2:** Autoimmune screen: positive for AQP4 IgG suggesting neuromyelitis optica spectrum disorder ANCA: antineutrophil cytoplasmic antibody; MOG: myelin oligodendrocyte glycoprotein; AQP4: aquaporin-4 antibody

Parameter	Results	Units	Reference
Erythrocyte sedimentation rate	7	mm/hr	0-20
Antinuclear antibody	1:320 (nucleolar, speckled)		<1:80
ANCA	Negative		Negative
MOG IgG	Negative		Negative
AQP4 IgG	Positive		Negative

The patient was ultimately diagnosed with NMOSD and was promptly treated with pulsed methylprednisolone followed by intravenous tocilizumab, resulting in gradual improvement of her neurological deficits. Following discharge, she has been under regular multidisciplinary follow-up involving neurology, ophthalmology, physiotherapy, and occupational therapy at 2-3 monthly intervals. At follow-up, she remains relapse-free on maintenance tocilizumab, with no new lesions identified on interval MRI.

## Discussion

Vomiting is a common and usually benign clinical presentation with a broad differential diagnosis, encompassing infectious, gastrointestinal, metabolic, cardiac, drug-related, and neurological causes [1]. While many etiologies are self-limiting, certain conditions require urgent recognition and intervention, including bowel obstruction, mesenteric ischemia, diabetic ketoacidosis, brainstem stroke, and disorders associated with raised intracranial pressure [8]. Failure to identify these conditions early may result in significant morbidity and mortality.

Demyelinating diseases of the central nervous system represent an important yet frequently under-recognized cause of persistent or intractable vomiting, particularly as neurological manifestations may evolve insidiously and appear later in the disease course. Disorders within this group include MS, NMOSD, MOGAD, and acute disseminated encephalomyelitis (ADEM) [8-9]. As a result, patients may initially undergo repeated gastrointestinal evaluations before a neurological etiology is considered, contributing to diagnostic delay.

NMOSD occurs more commonly in females, with a median age of onset between 32 and 41 years, and predominantly affects the optic nerves and spinal cord, with less frequent involvement of the brainstem [10]. When the brainstem is involved, patients may develop area postrema syndrome. The area postrema, located in the dorsal medulla, plays a critical role in the vomiting reflex and is particularly susceptible in NMOSD due to the absence of a blood-brain barrier and high expression of aquaporin-4 (AQP4) water channels [11]. Consequently, patients, especially those with AQP4-IgG seropositivity, may present with intractable hiccups, nausea, and vomiting, which may precede more classical neurological manifestations such as optic neuritis or transverse myelitis.

Our patient fulfilled the diagnostic criteria for NMOSD based on her clinical presentation with brainstem involvement, characteristic neuroimaging findings, and seropositivity for AQP4 immunoglobulin G (AQP4-IgG). Distinctive imaging features of NMOSD include longitudinally extensive spinal cord lesions and involvement of brain regions with high AQP4 expression. As demonstrated in this case, anatomical involvement of different regions of the central nervous system results in varied clinical manifestations; brainstem involvement led to cranial nerve dysfunction, impaired balance, and persistent nausea and vomiting [12]. The predilection of NMOSD for the area postrema reflects its high AQP4 expression, rendering this region uniquely vulnerable in this disease subtype [13].

NMOSD, MOGAD, and MS share overlapping clinical features, most notably optic neuritis, which is a common presenting manifestation across all three conditions. However, these disorders can be differentiated based on characteristic clinical and radiological features. Optic neuritis in MS typically presents unilaterally, whereas NMOSD and MOGAD more commonly present with bilateral involvement. NMOSD preferentially affects regions of the central nervous system with high AQP4 expression, while MS predominantly involves cerebral white matter, and MOGAD more frequently affects grey matter structures [14].

Spinal cord involvement also differs between these conditions. NMOSD is characteristically associated with longitudinally extensive, continuous cervical spinal cord lesions, while MS typically demonstrates multiple short-segment lesions. In contrast, MOGAD more commonly involves the lower thoracic spinal cord or conus medullaris [14]. These key distinguishing features are summarized in Table 3 [12].

**Table 3:** Differences between NMOSD, MOGAD, and MS NMOSD: neuromyelitis optica spectrum disorder; MOGAD: myelin oligodendrocyte glycoprotein antibody-associated disease; MS: multiple sclerosis; AQP4: aquaporin-4; ADEM: acute disseminated encephalomyelitis; MRI: magnetic resonance imaging

Feature	NMOSD	Multiple sclerosis	MOGAD
Typical age of onset	32-41 years	20-40 years	Young adults and children
Sex predilection	Female	Female	Nil
Key antibody	AQP4-IgG-positive	Seronegative	MOG-IgG positive
Initial presentation	Optic neuritis, transverse myelitis, and area postrema syndrome	Optic neuritis, sensory deficits, and motor weakness	Optic neuritis, ADEM-like illness
Optic neuritis	Often bilateral	Often unilateral	Often bilateral
Brain involvement	Areas of high AQP4 expression	Predominantly white matter	Predominantly grey matter
Brainstem involvement	Common	Uncommon	Rare
Vomiting/hiccups	Common	Rare	Uncommon
Spinal cord involvement	Longitudinally extensive transverse myelitis, commonly in the cervical region	Short segment, patchy lesions	Often lower thoracic or conus involvement
MRI lesions characteristics	Long, continuous lesions	Multiple discrete lesions	Poorly demarcated lesions
Relapse pattern	Relapsing with stepwise disability	Relapsing remitting	Relapsing with better recovery
Response to multiple sclerosis treatment	May worsen the disease	Beneficial	Variable
Long-term treatment	Immunosuppression	Disease-modifying therapies	Immunosuppression

Wernicke’s encephalopathy was another important differential diagnosis, as it classically presents with a triad of ophthalmoplegia, ataxia, and confusion secondary to thiamine deficiency [15]. It is commonly seen in individuals with chronic alcohol use or nutritional deficiency, which was a consideration in this case, given the patient’s one-month history of reduced oral intake and significant weight loss. The pattern of involvement on imaging also differs from NMOSD, which usually affects the medial thalami, mammillary bodies, and periaqueductal gray matter. A trial of intravenous thiamine administered early during admission did not result in clinical improvement, making this diagnosis less likely [16].

Management of NMOSD involves acute treatment with high-dose intravenous corticosteroids, with escalation to plasmapheresis in refractory cases, followed by long-term immunosuppressive therapy to reduce relapse risk and prevent irreversible neurological disability [17]. Early recognition of atypical presentations, such as intractable vomiting due to area postrema involvement, is therefore essential to facilitate timely diagnosis and improve long-term outcomes.

## Conclusions

Although vomiting is often benign and self-limiting, persistent, unexplained, or recurrent symptoms should prompt consideration of serious underlying causes. Neurological etiologies, particularly demyelinating disorders involving the brainstem, are an important but frequently under-recognized cause of intractable vomiting. Area postrema syndrome may represent the earliest manifestation of NMOSD and can precede classic features such as optic neuritis or transverse myelitis. Failure to recognize vomiting as a neurological red flag can lead to repeated hospital presentations, unnecessary gastrointestinal investigations, and delays in neuroimaging, specialist referral, and immunotherapy. This case underscores the importance of a broad differential diagnosis, multidisciplinary evaluation, and early recognition to prevent irreversible neurological injury and improve patient outcomes.
